# A study of depression, partnership and sexual satisfaction in patients with post-traumatic olfactory disorders

**DOI:** 10.1038/s41598-021-99627-9

**Published:** 2021-10-12

**Authors:** Seyed Kamran Kamrava, Zeinab Tavakol, Atefeh Talebi, Mohammad Farhadi, Maryam Jalessi, Seyedeh Fahimeh Hosseini, Elahe Amini, Ben Chen, Thomas Hummel, Rafieh Alizadeh

**Affiliations:** 1 ENT and Head & Neck Research Center and Department, The Five Senses Health Institute, Hazrat Rasoul Akram Hospital, Iran University of Medical Sciences, Tehran, Iran; 2 Community-Oriented Nursing Midwifery Research Center, Nursing and Midwifery School, Shahrekord University of Medical Sciences, Shahrekord, Iran; 3 Colorectal Research Center, Iran University of Medical Sciences, Tehran, Iran; 4 Skull Base Research Center, The Five Senses Health Institute, Hazrat Rasoul Akram Hospital, Iran University of Medical Sciences, Tehran, Iran; 5 Medical Physics Department, School of Medicine, Iran University of Medical Sciences, Tehran, Iran; 6 Department of Geriatric Psychiatry, The Affiliated Brain Hospital of Guangzhou Medical University (Guangzhou Huiai Hospital), Guangzhou, China; 7 Department of Otorhinolaryngology, Smell and Taste Clinic, Technische Universität, Dresden, Germany

**Keywords:** Outcomes research, Neuroscience, Anatomy, Medical research

## Abstract

Post-traumatic olfactory dysfunction (PTOD) is associated with a significant decrease in quality of life. The present study aimed to explore whether PTOD is associated with depression and changes in sexuality. There were two groups in this case–control study. The patient group consisted of patients with PTOD (n = 55), and the control group comprised healthy individuals without the olfactory disorder (n = 115). Olfactory function, depression, partnership, and sexual satisfaction were assessed using the Iranian version of the Sniffin’ Sticks test (Ir-SST), Beck Depression Inventory (BDI), Enrich Couple Scale (ECS) and Sexual Satisfaction Scale for Women (SSSW). The BDI scores were higher in the patient group than in the control group (p < 0.001). The SSSW score was lower in the patient group than in controls (p < 0.01), although the ECS score was not significantly different between patients and controls. Also, there was no significant difference in the severity of trauma between marital satisfaction and sexual satisfaction. However, the analysis showed a statistically significant difference in depression scores in connection with the head trauma severity. In the PTOD group, depression was increased and sexual satisfaction declined. Understanding the association of olfactory dysfunction with depression and sexuality allows patients and doctors to deal with less notable consequences of this disorder.

## Introduction

Olfactory dysfunction affects about 20% of the population^1^. Apart from aging, the most common causes of olfactory disorder are chronic rhinosinusitis, upper respiratory tract infections and head trauma^2–4^. Post-traumatic olfactory dysfunction (PTOD) may result in either complete (anosmia) or incomplete (hyposmia) loss of smell^5–7^. The presence of PTOD varies widely from 4 to 65% in studies^8–10^.

An association between head trauma severity and the severity of olfactory loss has been reported^11,12^. However, mild head trauma may also be linked to complete olfactory loss^10,13^.

Olfactory dysfunction has a strong impact on the life of patients and may give rise to a plethora of problems^14^. However, despite its importance, the effect of this olfactory dysfunction on patients’ life is often overlooked. Many studies have attempted to explore the effects of olfactory dysfunction on different aspects of life. For example, in the last two decades, the association between olfactory dysfunction and depression has been studied^15–17^. Still, the exact relationship between olfactory dysfunction and depression is not entirely known^18^.

Other important aspects of an individual’s life are marital satisfaction and sexuality. Marital or partnership satisfaction is a multi-factorial construct^19^ that can serve as an indirect measure for the overall quality of life. Olfaction is important for human sexual life^20^. The rate of divorce or separation after head trauma is reported to be in the range of 15% to 78%^21–23^. The effect of olfactory dysfunction on sexual life does not appear immediately and could be mediated by a number of concomitant variables. In a sample of patients with olfactory disorders, Gudziol et al.^24^ observed that sexual problems were not directly associated with olfactory function. Rather, they were linked to the depression symptoms caused by olfactory loss. Previous research has shown a bi-directional link between olfactory impairment and depression^25^, as olfactory and emotional processes are in overlapping brain structures and any alterations in one network can affect the other domains.

Therefore, PTOD could be linked to major adverse consequences^26^. Hence, the goal of this case–control study was to examine the impact of olfactory loss induced by head trauma on depression, marital/partnership, and sexual satisfaction in the Iranian population.

## Methods

### Ethics statement

This research complies with the principles of the Helsinki Declaration. Written informed consent was obtained from patients and healthy subjects before the administration of tests. All experimental protocols was approved by the Research Ethics Committee of the Iran University of Medical Sciences (Ethical code: IR.IUMS.REC 1396.3137). Since the participants had only experienced head trauma without any particular brain problems, and were fully conscious, we managed to obtain informed written consent from all participants before the study.

### Participants

There were two groups in this case–control study. The patient group consisted of patients with PTOD lasting 2 to 36 months (n = 55), and the control group includes healthy individuals without an olfactory disorder (n = 115). Patients were referred to the ENT Research Center of Rasoul-e Akram Hospital affiliated with the Iran University of Medical Sciences between July 2017 and December 2018. The control group consisted of healthy companions of the patients.

Inclusion criteria were being married, having regular sex with one’s partner, and 20–60 years of age. The exclusion criteria in both groups were congenital anomalies (isolated congenital anosmia or Kallmann’s Syndrome), nasal septum deviation, neurological diseases such as Parkinson's or Alzheimer's disease, diabetes, smoking, current upper respiratory tract infection (URTI), sinonasal disease, and pregnancy.

### Study design

At the outset of the study, two groups (patients with PTOD, healthy controls) were selected. Then, the olfactory performance of the patients was measured to determine the severity of olfactory malfunction. Olfactory tests were also run in healthy individuals to determine normosmia. In the next step, olfactory function, depression, partnership, and sexual satisfaction were assessed using the Iranian version of the “Sniffin’ Sticks” test (Ir-SST), Beck Depression Inventory (BDI), Enrich Couple Scale (ECS) and Sexual Satisfaction Scale for Women(SSSW) in both groups.

The participants in the study group (PTOD) was divided into 3 groups based on the severity of trauma (mild, moderate and severe) as follows:

- Mild: Patients treated on an outpatient basis without a brain injury
- Moderate: Patients admitted to Intensive Care Unit (ICU) without a brain injury
- Severe: ICU patients with a brain injury

### Olfactory performance assessment

The olfactory function was assessed in all participants using the Ir-SST, which comprises three subtests: (1) odor threshold for phenyl ethyl alcohol (PEA; single staircase, 3 alternative-forced choice tasks), (2) odor discrimination (16 triplets of odors, 3 alternative-forced choice tasks), and (3) odor identification (16 common odorants, forced-choice identification from four verbal descriptors of each odor)^27^.

The overall performance score on the Sniffin’ Sticks test (the sum of results from threshold testing with a score of 1–16; odor discrimination with a score of 0–16, and odor identification with a score of 0–16, designated as 1–48) was used as the for patients in three diagnostic groups: normosmia, hyposmia, and functional anosmia. A TDI score basis (Threshold, Discrimination, Identification) smaller than 16.5 indicates functional anosmia, a score of 16.5 to 30.5 denotes hyposmia and a score of above 30.5 suggests normosmia. A clinical interview by a physician assessed the duration of smell loss and the etiology of olfactory dysfunction^27^.

### Questionnaires

The demographic information of participants was collected by a questionnaire that asked questions about sex, age, education level, job status, and marriage. To evaluate the severity of depression, the patients filled out the Iranian version of the Beck Depression Inventory (BDI), which consists of 21 multiple-choice items. In this inventory, a score of 0–10 implies the absence of depression, 11–30 denotes mild depression, and 31–105 suggests severe depression^28,29^. Marital satisfaction was also measured by the Iranian version of the Enrich Couple Scale^30^ which consists of 35 items scored on a 5-point Likert scale. In this scale, a score of 0–80 shows marital dissatisfaction, 81–120 partial satisfaction, and 121–180 complete satisfaction. Finally, sexual satisfaction was measured by the Iranian version of the Sexual Satisfaction Scale for Women(SSSW), which includes 30 items rated on a5-point Likert scale. In this questionnaire, a score of 0–50 implies sexual dissatisfaction, 51–100 shows partial satisfaction and 101–150 reflects complete satisfaction^31^. The Cronbach's alpha of the Enrich Couple Scale, the Beck Depression Inventory (BDI) and the Sexual Satisfaction Scale for Women(SSSW) was 0.899, 0.913 and 0.959 respectively.

### Statistical analysis

Descriptive statistics such as mean ± SD were used to describe results as continuous variables. In addition, the frequency and percentage of categorical variables were reported. The normality of continuous variables was assessed by the Kolmogorov–Smirnov test. Moreover, the independent T-test was used to compare mean age in groups with different levels of TDI scores. Furthermore, this test was run to compare the mean of marital satisfaction, sexual satisfaction and depression in both case and control groups. The analysis of variance (ANOVA) was conducted to compare the mean values of depression, marital satisfaction and sexual satisfaction in three categories of trauma severity. The linear regression was applied to predict the effect of dependent variables, including depression, marital satisfaction, sexual satisfaction and independent variables such as age, sex, duration of olfactory disorder, the severity of the trauma, and education in participants. In addition, a partial correlation was performed to discover the relationship between TDI scores and depression in patients while controlling the effect of marital satisfaction and sexual satisfaction. A significance level of 0.05 was considered for this study. The statistical analyses were performed using SPSS 24 software. The sample size was estimated based on these assumptions: a large expected effect size (~ 0.8), an alpha level of 0.05, test power of 0.95, and allocation ratio (patients/controls) of 1:2. Finally, a sample of n = 94 subjects was obtained (n = 31 in the patient group, and n = 63 in controls).

## Results

As expected, the TDI score of 55 individuals in the patient group (PTOD) was below 30.5 (29 females and 26 males; 25–63 years of age with a mean age of 39.8 ± 9.11 years) and 115 subjects in the control group had a TDI score greater than 30.5 (60 females and 55 males; 23–53 years of age with a mean age of 37.7 ± 8.08 years). The mean ± SD and the range of TDI were 23.5 ± 7.9, 8–29.7 in the patient group and 36.3 ± 5.6, 32.4–47.1 in the control group. The two groups were matched for age (p = 0.16). A significant difference was observed in gender distribution between the two groups (p < 0.001) so that there were more men in the PTOD group.

**Figure 1:**
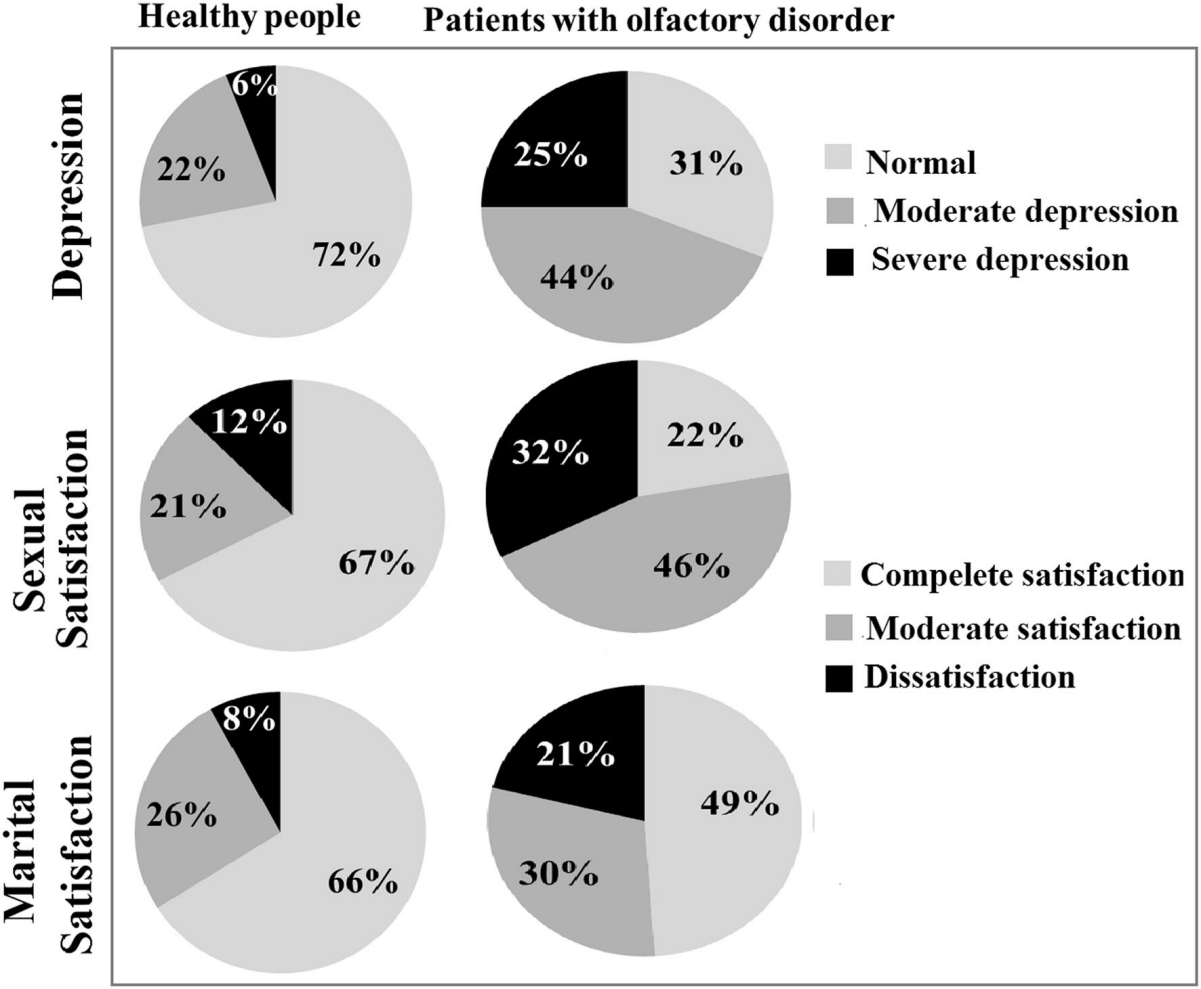
Frequency of depression, sexual and marital satisfaction in patients and controls.

The frequency of depression in the control and patient group is shown in Fig. 1. The mean scores of depression were significantly higher in the patient group than in the healthy group (p < 0.001) (Table 1). In addition, mean scores of depression were significantly higher in the anosmia group than in the hyposmia group (26 ± 2.32 vs. 11.6 ± 2.05, respectively), (p = 0.009).

**Table 1:** Mean score of depression, sexual and marital satisfaction in patients and healthy controls.

Variable	Controls	Patients	(Max–min)	Test statistics	p
Age	37.7 ± 8.08	39.86 ± 9.11	(63–23)	T = 1.42	p = 0.18
Depression	8.64 ± 2.1	21.75 ± 3.41	(50–0)	T = 5.49	p < 0.001
Sexual satisfaction	115.5 ± 37.05	54 ± 8.07	(120–43.5)	T = 2.54	p = 0.012
Marital satisfaction	144.33 ± 12.99	96.4 ± 10.77	(160–62)	T = 0.084	p = 0.926
**Sex**			–	${\chi }^{2}$ = 60.99	p < 0.001
Male	40	16			
Female	15	99			

The frequency of sexual satisfaction in both groups is depicted in Fig. 1. The mean score of sexual satisfaction was significantly lower in the patient group than in controls (p = 0.012) (Table 1), but the mean score of marital satisfaction was not significantly different than the patient group.

The frequency of marital satisfaction in both groups is illustrated in Fig. 1. The mean marital score was not significantly different between the two groups (Table 1).

**Figure 2:**
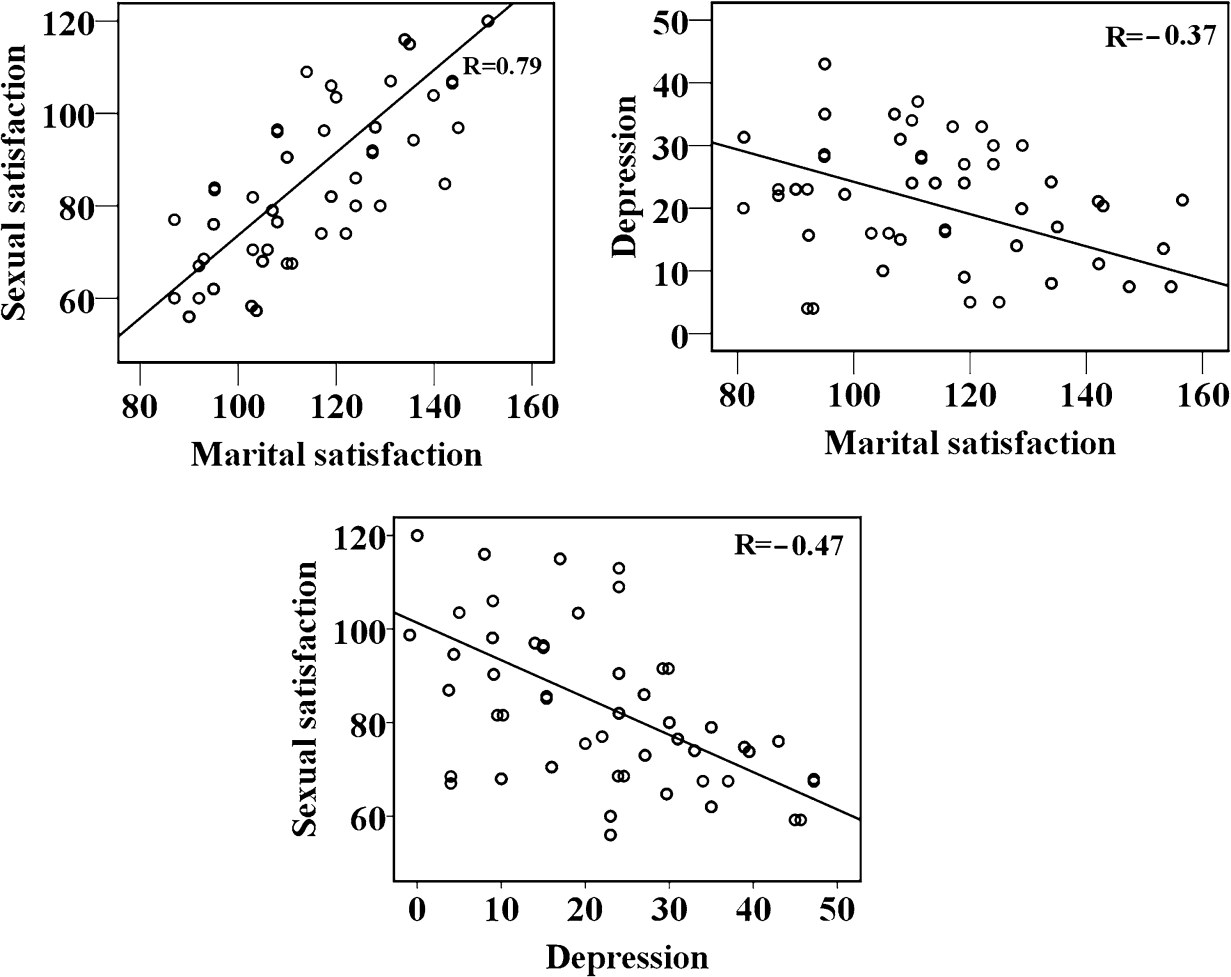
Scatter plots for correlations in the patient group between sexual satisfaction, depression, and marital satisfaction.

Higher marital satisfaction was associated with higher sexual satisfaction (r = 0.79, p < 0.001), and lower symptoms of depression (r = 0.37, p < 0.001) (Fig. 2). In addition, results showed that more severe depression was linked to lower levels of sexual satisfaction (r = − 0.47 p < 0.001) (Fig. 2).

**Figure 3:**
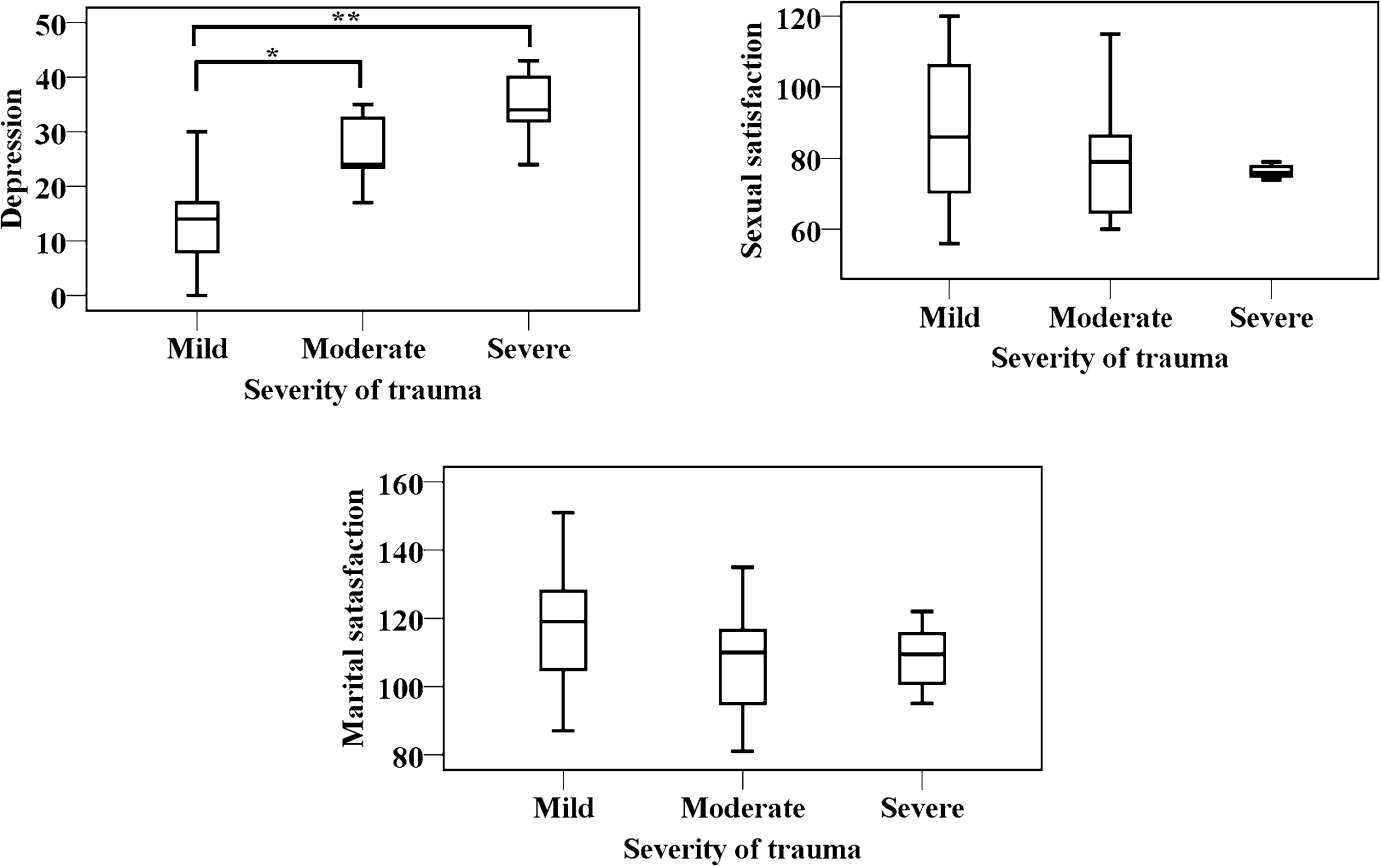
Box plot of differences between sexual satisfaction, depression, and marital satisfaction, in relation to the severity of trauma with in the patient group (n = 55).

Figure 3 shows Box plots that display the relationship of the severity of trauma with depression, sexual and marital satisfaction of patients. The result of the ANOVA test revealed the lack of a significant difference in the severity of trauma between marital and sexual satisfaction. However, the analysis demonstrated a significant difference in depression scores in connection with head trauma severity (p = 0.04). The results of Bonferroni adjusted alpha level showed differences between mild head trauma and moderate/severe head trauma(p < 0.05/3).

According to Table 2, the severity of trauma had a significant effect on depression (B = 26.5, B = 11.65, p < 0.001), meaning that depression scores are 26.5 units lower in patients with mild head trauma than in patients with severe head trauma. Moreover, the depression score in patients with moderate head trauma was 11.65 units lower than in patients with severe head trauma.

**Table 2:** Multivariable regression predicting sexual and marital satisfaction and depression.

Variables		B	Std. error	Beta	T-value	p-value	95% CI for B	
Depression	**Severity**							
	Severe (reference)	–	–	–	–	–	–	–
	Mild	− 26.495	3.910	− 1.119	− 6.776	< 0.001***	− 34.450	− 18.540
	Moderate	− 11.646	4.429	− 0.453	− 2.630	0.01**	− 20.657	− 2.636
	**Sex**	− 4.188	3.052	− 0.154	− 1.372	0.179	− 10.397	2.021
	**Age**	− 0.117	0.158	− 0.085	− 0.739	0.71	− 0.437	0.204
Sexual satisfaction	**Severity**							
	Severe (reference)	–	–	–	–	–	–	–
	Mild	14.652	5.871	0.378	2.493	0.010**	2.759	26.557
	Moderate	2.506	10.931	0.059	0.229	0.763	− 19.734	24.746
	**Sex**	10.393	7.533	0.232	1.380	0.177	− 4.933	25.719
	**Age**	− 0.280	0.389	− 0.124	− 0.721	0.201	− 1.072	0.511
Marital satisfaction	**Severity**							
	Severe (reference)	–	–	–	–	–	–	–
	Mild	5.852	8.340	0.180	0.702	0.259	− 11.115	22.819
	Moderate	0.128	9.446	0.004	0.014	0.826	− 19.089	19.346
	**Sex**	− 3.918	5.776	− 0.104	− 0.678	0.501	− 15.574	7.739
	**Age**	− 0.545	0.336	− 0.289	− 1.621	0.123	− 1.229	0.139

**p < 0.05, Sexual satisfaction in mild trauma vs. severe trauma.

***p < 0.001, Depression in mild trauma vs. sever trauma.

Similarly, the sexual satisfaction score was higher in patients with mild trauma than in patients with severe head trauma (B = 14.6, p = 0.01). This suggests that the sexual satisfaction score was 14.6 units higher in patients with mild head trauma than in patients with severe head trauma.

## Discussion

The results reveal that depression is significantly higher and sexual satisfaction is diminished in PTOD patients.

According to the results, more than 65% of participants in the patient group had high depression scores. Philpott et al. reported severe depression (43%) and relationship difficulties (54%) in patients with olfactory disorders^32^. Also, Merkonidis et al. stated that 36% of participants with an olfactory disorder were in a depressed mood^33^. The rationale behind the higher percentage of depression in patients with olfactory disorders is not exactly known, but it may be related to the overlap between brain areas that process olfactory and emotional information. PTOD is typically accompanied by damage to the central nervous system, especially in the olfactory bulb and frontal lobe^34^. Since the olfactory system is strongly linked to the emotional network, and both the olfactory bulb and frontal lobe are involved in emotion regulation, damage to odor-related brain areas may explain the rise of depression symptoms.

In addition, the association of olfactory function with mood, eating, and social relations seems to favor depression symptoms in cases of olfactory loss^35,36^. In particular, olfactory disorder induces changes in olfactory-related tasks (e.g., at the workplace, during meal times, social communications, maintenance of hygiene standards or detection of environmental hazards). This can affect the quality of life and magnify the symptoms of depression.

In some cases, PTOD patients complain about their impaired sexual life^20^. The results of this study demonstrated that more than 75% of subjects in the patient group exhibited decreased sexual satisfaction. This change was predicted by depression symptoms and olfactory function. Merkonidis et al. observed that people with olfactory disorders confront problems in their sexual life. In their survey, more than half of patients (57%) reported changes in sexuality^33^.

In a sample of patients with smell disorders, Gudziol et al.^24^ revealed that sexual problems are not directly associated with the level of olfactory function, but with depression symptoms induced by the olfactory disorder. Considering that patients are typically unwilling/unprepared to report those intimate issues, healthcare professionals must pay heed to this common side effect by asking direct questions about the sexual relations of patients.

The olfactory disorder may influence sexual satisfaction directly or indirectly by changing mood and/or happiness. In this regard, the perception of body odors during sexual intercourse is assumed to contribute to sexual arousal^37,38^, and therefore is an integral part of sexual satisfaction. These relatively subtle effects may modify the willingness to leave or stay in an intimate relationship^20^. It is worth noting that impairments in intimate affairs such as sexual relations are inherently associated with shame and embarrassment, which poses major barriers to treatment seeking. Hence, when counseling patients with PTOD, the patients’ sexuality should be actively discussed during the interview.

In our study, more than 50% of participants in the study group reported decreased marital satisfaction. Overall, however, no significant difference was observed between patients with the olfactory disorder and healthy controls in terms of marital satisfaction. Apart from this, sexual satisfaction in the patient group was significantly different from healthy controls. This indicates that sexual satisfaction is only one of several dimensions of marital satisfaction. Marital and sexual satisfaction protects people against psychological distress but could also be related to depression^39,40^. The present results may also be biased in terms of social barriers to reporting marital problems.

Regarding the severity of the trauma, it was observed that patients with severe trauma experienced a significantly higher level of depression and lower level of sexual satisfaction compared to patients with mild to moderate head trauma. The severity of head trauma in patients with PTOD was also found to be a significant predictor of depression and sexual satisfaction. This was supported by the result of regression analysis showing that the severity of trauma could be regarded as an important predictor for symptoms of depression and sexual satisfaction. However, given the relatively small numbers of participants in the subgroups, caution should be exercised in interpreting the results of regression analysis.

In our results, severe depression is associated with lower marital and sexual satisfaction while great sexual satisfaction is linked to higher marital satisfaction in these patients. To discover causal factors between the affected variables, further longitudinal research with larger sample sizes is warranted. Based on the study data, it is suggested to raise awareness of the impact of olfactory dysfunction on mental health and sexual life in routine care settings.

## Limitations

The present study had several limitations that should be considered when interpreting the results. First, this study was conducted only in one research center, which hampers the generalizability of its results. Second, the self-reporting questionnaire used for data collection in order to measure depression, partnership and sexual satisfaction may be a source of bias. Third, comparing two demographically similar groups that are mainly different in terms of self-reported olfactory may undermine the impact of this bias. Forth, it is suggested that future studies include controls that are not associated with the patients—as was the case in the present study, to eliminate this source of bias. Besides, the inclusion of the patients’ companions as a control group ensures comparable social and environmental backgrounds. Fifth the cross-sectional nature of the study design constrains inferences about the causal relationships between olfactory dysfunction and other variables (depression, sexual and marital satisfaction). Longitudinal research can help shed further light on this relationship, as such relationships are multi-factorial and complex.

## Conclusion

Post-traumatic olfactory dysfunction is associated with impaired sexual life and increased depression symptoms. The results of this study can help provide more comprehensive services to the patients. Also, the results highlight the fact that healthcare settings should be more observant of the potential side effects associated with olfactory loss and ask explicit questions regarding the special aspects of this impairment in a bid to lower the barriers that inhibit patients from seeking proper assistance.
